# The Metabolic and Non-Metabolic Roles of UCK2 in Tumor Progression

**DOI:** 10.3389/fonc.2022.904887

**Published:** 2022-05-20

**Authors:** Yi Fu, Xin-dong Wei, Luoting Guo, Kai Wu, Jiamei Le, Yujie Ma, Xiaoni Kong, Ying Tong, Hailong Wu

**Affiliations:** ^1^Affiliated Zhoupu Hospital, Shanghai University of Medicine and Health Sciences, Shanghai, China; ^2^Shanghai Key Laboratory of Molecular Imaging, Collaborative Innovation Center for Biomedicines, Shanghai University of Medicine and Health Sciences, Shanghai, China; ^3^Central Laboratory, Department of Liver Diseases, Institute of Clinical Immunology, ShuGuang Hospital Affiliated to Shanghai University of Traditional Chinese Medicine, Shanghai, China; ^4^School of Pharmacy, Shanghai University of Medicine and Health Sciences, Shanghai, China; ^5^Department of Liver Surgery, Renji Hospital, School of Medicine, Shanghai JiaoTong University, Shanghai, China

**Keywords:** UCK2, chemotherapy, pyrimidine salvage synthesis, tumor development and progression, non-metabolic role of UCK2, oncogene, cytotoxic ribonucleoside analogs

## Abstract

Enhanced nucleoside metabolism is one of the hallmarks of cancer. Uridine-cytidine kinase 2 (UCK2) is a rate-limiting enzyme of the pyrimidine salvage synthesis pathway to phosphorylate uridine and cytidine to uridine monophosphate (UMP) and cytidine monophosphate (CMP), respectively. Recent studies have shown that UCK2 is overexpressed in many types of solid and hematopoietic cancers, closely associates with poor prognosis, and promotes cell proliferation and migration in lung cancer and HCCs. Although UCK2 is thought to catalyze sufficient nucleotide building blocks to support the rapid proliferation of tumor cells, we and other groups have recently demonstrated that UCK2 may play a tumor-promoting role in a catalytic independent manner by activating oncogenic signaling pathways, such as STAT3 and EGFR-AKT. By harnessing the catalytic activity of UCK2, several cytotoxic ribonucleoside analogs, such as TAS-106 and RX-3117, have been developed for UCK2-mediated cancer chemotherapy. Moreover, we have demonstrated that the concurrent targeting of the catalytic dependent and independent features of UCK2 could synergistically inhibit tumor growth. These findings suggest that UCK2 may serve as a potential therapeutic target for cancer treatment. In this mini-review, we introduced the genomic localization and protein structure of UCK2, described the role of UCK2 in tumor development, discussed the application of UCK2 in anti-tumor treatment, and proposed concurrent targeting of the catalytic and non-catalytic roles of UCK2 as a potential therapeutic strategy for cancer treatment.

## Introduction

As early as 1926, Warburg proposed that altered cellular metabolism is essential for the uncontrolled growth of cancer (1). Among those metabolism alterations, a constant and sufficient supply of nucleotides is required for the rapid proliferation of cancer cells (2). There are two pathways for nucleotide synthesis: *de novo* and salvage (3). The *de novo* synthesis starts with building materials such as amino acids, ribose-5-phosphate, CO_2_, and NH_3_, whereas the salvage synthesis utilizes recycled free bases and nucleosides (3). It has been generally accepted that the rapid proliferating cells favor the *de novo* synthesis pathway, whereas the differentiated cells mainly rely on the less energy requiring salvage pathway (4). However, a recent study demonstrated that the utilization of salvage pathway and *de novo* pathway for purine synthesis was comparable in colorectal carcinoma (CRC) (5), suggesting that the salvage pathway may also play an important role in cancer development and progression. Interestingly, the salvage pathway for pyrimidine synthesis is more efficient than that for purine synthesis (3), but the role of the pyrimidine salvage synthesis in tumor development is largely unknown.

**Figure 1:**
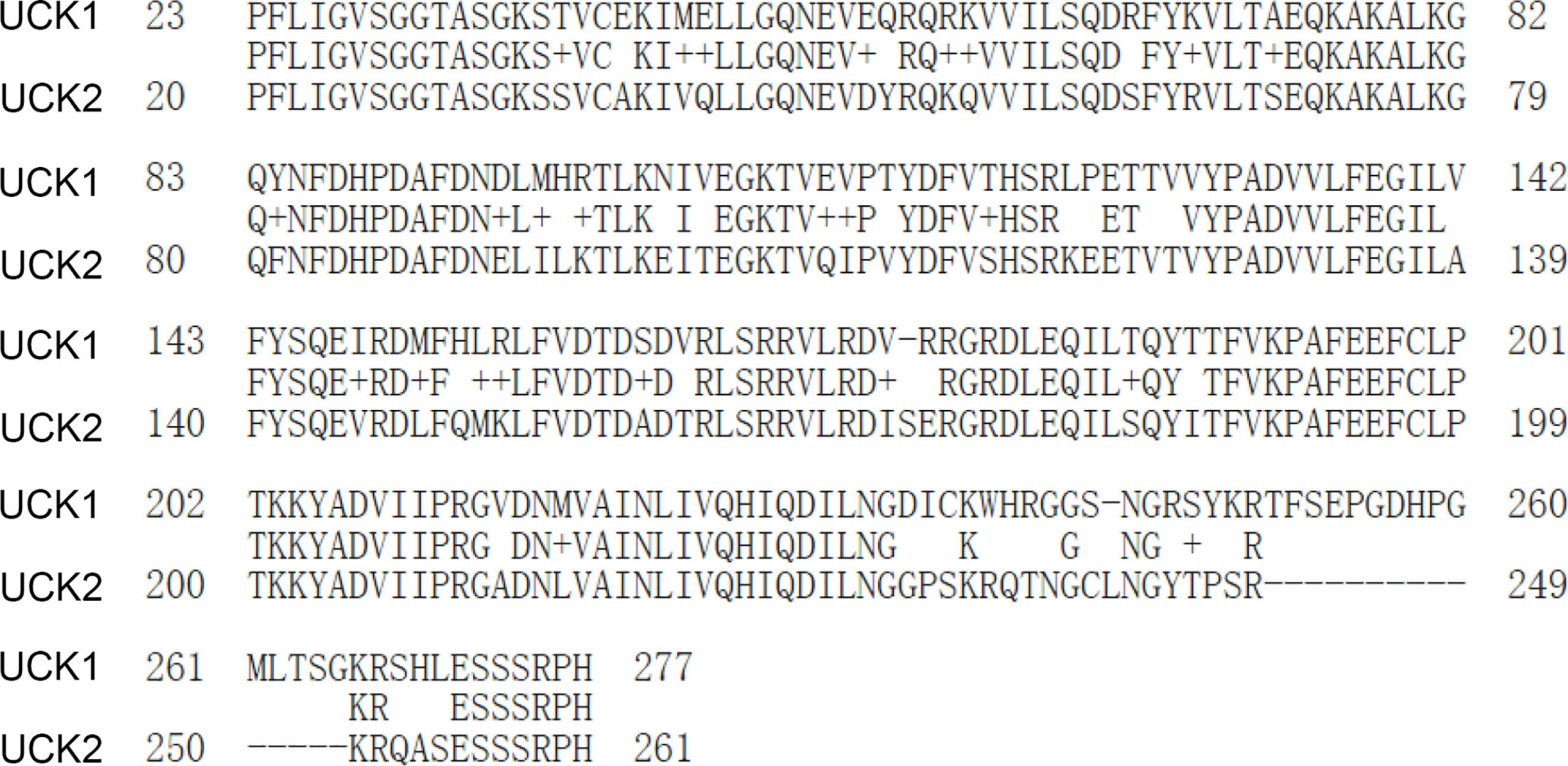
The alignment of the protein sequences of UCK1 and UCK2.

Uridine-cytidine kinases (UCKs) are the rate-limiting enzymes of the pyrimidine salvage synthesis to phosphorylate uridine and cytidine to uridine monophosphate (UMP) and cytidine monophosphate (CMP), respectively (6). Human UCKs consist of two members: UCK1 and UCK2 (6). Although human UCK1 and UCK2 proteins share 72% sequence identity (**Figure 1**), the catalytic efficiency of UCK2 is 15–20 times higher than that of UCK1 (7). Meanwhile, when UCK1 expresses universally in various tissues, UCK2 exclusively expresses in human placenta and several types of cancers (6, 8). In this mini-review, we introduced the genomic localization and protein structure of UCK2, described the role of UCK2 in tumor development, and discussed the application of UCK2 in anti-tumor treatment.

## Genomic Localization and Protein Structures of UCK2

As early as the 1970s, Medrano and Green had provisionally assigned human *UCK2* to chromosome 1 by using somatic cell hybrids between a uridine kinase-deficient mouse cell line and human cells (9, 10). In 2001, human *UCK2* was mapped to chromosome 1q22-23.2 (7). Human *UCK2* spans more than 19 kb, carries seven exons, and encodes a 261–amino acid protein peptide with a predicted molecular mass of 29 kDa (7). Because of the sequence homology, UCK2 has been suggested to belong to the nucleoside kinases of nucleoside monophosphate (NMP) kinase fold family (11). However, unlike other NMP family members, who function as monomers, lack catalytic specificity for the sugar moiety of their substrates, and are not subjected to feedback inhibition, UCK2 functions as a homotetramer, specifically recognizes ribonecleosides but not 2’-deoxyribonecleosides, and is subjected to UTP- and CTP-mediated feedback inhibition (11–13). In addition, according to protein structural homology, Suzuki et al. further subgrouped UCK2 into UCK family with other two non-nucleic acid kinases, pantothenate kinase, and phosphoribulokinase (11).

**Figure 2:**
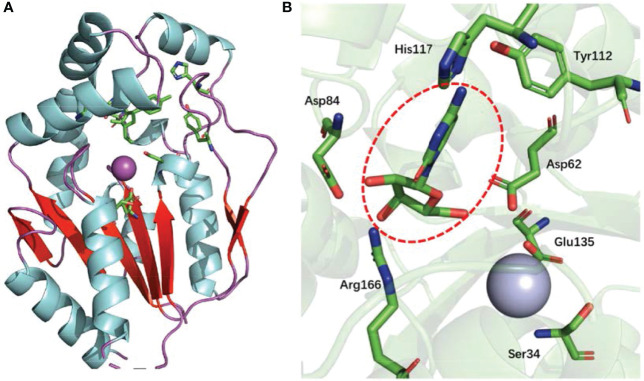
Protein structure of UCK2 and key amino acids for UCK2’s catalytic activity. **(A)** Ribbon diagram of human UCK2 monomer (PDB code 1UEJ), illustrating binding of cytidine to the active site. The purple sphere indicates a magnesium ion in the active site. The core five-stranded parallel β-sheet and the β-hairpin loop are labeled in red. **(B)** Stereo view of the active site of UCK2 showing a central bound cytidine (circled with red dashed line). A magnesium ion (cryan sphere) is coordinated by the Asp62, Ser34, and Glu135 at active site.

The UCK2 monomer contains a core five-stranded parallel β-sheet, which is flanked on both sides by two α-helices (α-helice flank) and capped by three α-helices (α-helice cap). In addition, a large β-hairpin loop sits nearby the core five-stranded parallel β-sheet. The enzyme active site of UCK2 locates at the region between the core five-stranded parallel β-sheet, the α-helice cap, and the large β-hairpin loop (11, 14) (**Figure 2A**). The nucleoside binding specificity of UCK2 is mainly determined by the His117 and Tyr112 residues, which form hydrogen bonds with the 4-amino group of cytidine or the 6-oxo group of uridine (14) (**Figure 2B**). Among those two residues, His117 is specific for uridine phosphorylation because the replacement of His117 with a Tyr residue resulted in a loss of uridine phosphorylation activity (15). In addition, Asp84 and Arg166 also form hydrogen bonds with the 2′- and 3′-hydroxyl groups of the ribose moiety which forces UCK2 to select ribonucleosides over deoxyribonucleosides substrates (14). In the UCK2-mediated catalytic reaction, Asp62 is responsible for attacking the γ-phosphorus of ATP by deprotonating and activating the 5′-hydroxyl group of the nucleoside substrates (14). In addition, the active site of UCK2 also contains one magnesium ion as a cofactor that is coordinated by the Asp62, Ser34, and Glu135 (14) (**Figure 2B**).

## Role of UCK2 in Tumor Development

Compared to the *de novo* nucleotide synthesis pathway, enzymes involved in the salvage synthesis pathway, especially for UCKs, displayed higher activities in the rapid growing hepatoma in parallel with the induction of neoplastic transformation (16), suggesting that they are important for tumor progression (17, 18). In fact, several lines of evidence have indicated the potential oncogenic role of *UCK2* (**Table 1**). First, upregulation of *UCK2* and/or increased UCK2 enzyme activity had been observed in many cancer types, including lung cancer (19, 20), breast cancer (21, 22), ovarian carcinoma (23), colorectal cancer (24), neuroblastoma (6, 8), pancreatic tumor (25), and hepatocellular carcinomas (HCCs) (26–29); second, *UCK2* was one of the most overexpressed genes in Burkitt lymphomas and acute lymphoblastic leukemia with gain of 1q (30); third, *UCK2* expression was positively associated with poor overall survival and disease-free survival in HCCs (27–29) pancreatic cancer (34) and lung cancer patients (20); fourth, a strong association between *UCK2* genetic variations and the risk of testicular germ cell tumors (TGCTs) was established through a meta-analysis of two independent genome-wide association studies (GWASs) (35); fifth, *UCK2* was one of the m^6^A RNA methylation-related genes, which is inversely related to the survival in patients with HCC (31); sixth, recent studies showed that *UCK2* is included in a macrophage-related four-gene signature or a six-gene prognostic signature that showed greater predictive performance for the OS in patients with HCC (32, 33); seventh, *UCK2* was included in a metabolism-related nine-gene signature and a cell cycle–related five-gene signature in distinguishing patients with high- and low-risk endometrial cancer (EC) and predict their OS (36, 37); eighth, a hypoxia-induced LncRNA-NEAT1 had been shown to increase *UCK2* expression and, in turn, result in enhanced HCC progression by serving as a sponge of miR-199a-3p (40). Although UCK2 is overexpressed in many cancer types, the The Cancer Genome Atlas (TCGA) pan-cancer analysis on UCK2 is missing. So far, no defined upstream factors have been reported to induce UCK2 expression in tumor. However, some previous studies do have indicated that the aberrant overexpression of UCK2 might be attributed to the gene amplication of UCK2 (27), the infection of Epstein–Barr virus (38), the m^6^A modification induced by METTL3 (39), hypoxia (40), and downregulation of certain miRNAs (46). Moreover, functional studies have demonstrated that overexpression of *UCK2* can promote cell proliferation and migration in lung cancer and HCCs (20, 27). Intriguingly, a recent study indicated that *UCK2* circular RNAs, circUCK2 (hsa_circ-001128 and hsa_circ_001357), could reduce cell proliferation and invasion in castration-resistant prostate cancer (CRPC) cells (47).

**Table 1:** UCK2 in tumor development and chemotherapy.

Features	Subjects	Ref.
UCK2 upregulation in	Lung cancer	(19, 20)
	Breast cancer	(21, 22)
	Ovarian carcinoma	(23)
	Colorectal cancer	(24)
	Neuroblastoma	(6, 8)
	Pancreatic tumor	(25)
	Hepatocellular carcinoma	(26–29)
	Burkitt lymphomas	(30)
	Acute lymphoblastic leukemia	(30)
UCK2 as a prognostic marker in	Hepatocellular carcinoma	(27–29, 31–33)
	Pancreatic cancer	(34)
	Lung cancer	(20)
	Testicular germ cell tumors	(35)
	Endometrial cancer	(36, 37)
Regulation of UCK2 expression by	Genomic amplification	(27)
	Infection of Epstein–Barr virus	(38)
	m6A modification	(39)
	Hypoxia	(40)
Cytotoxic ribonucleoside analogs of UCK2	TAS-106	(25, 41, 42)
	EUrd	(42)
	5-FU	(43)
	RX-3117	(44, 45)

**Figure 3:**
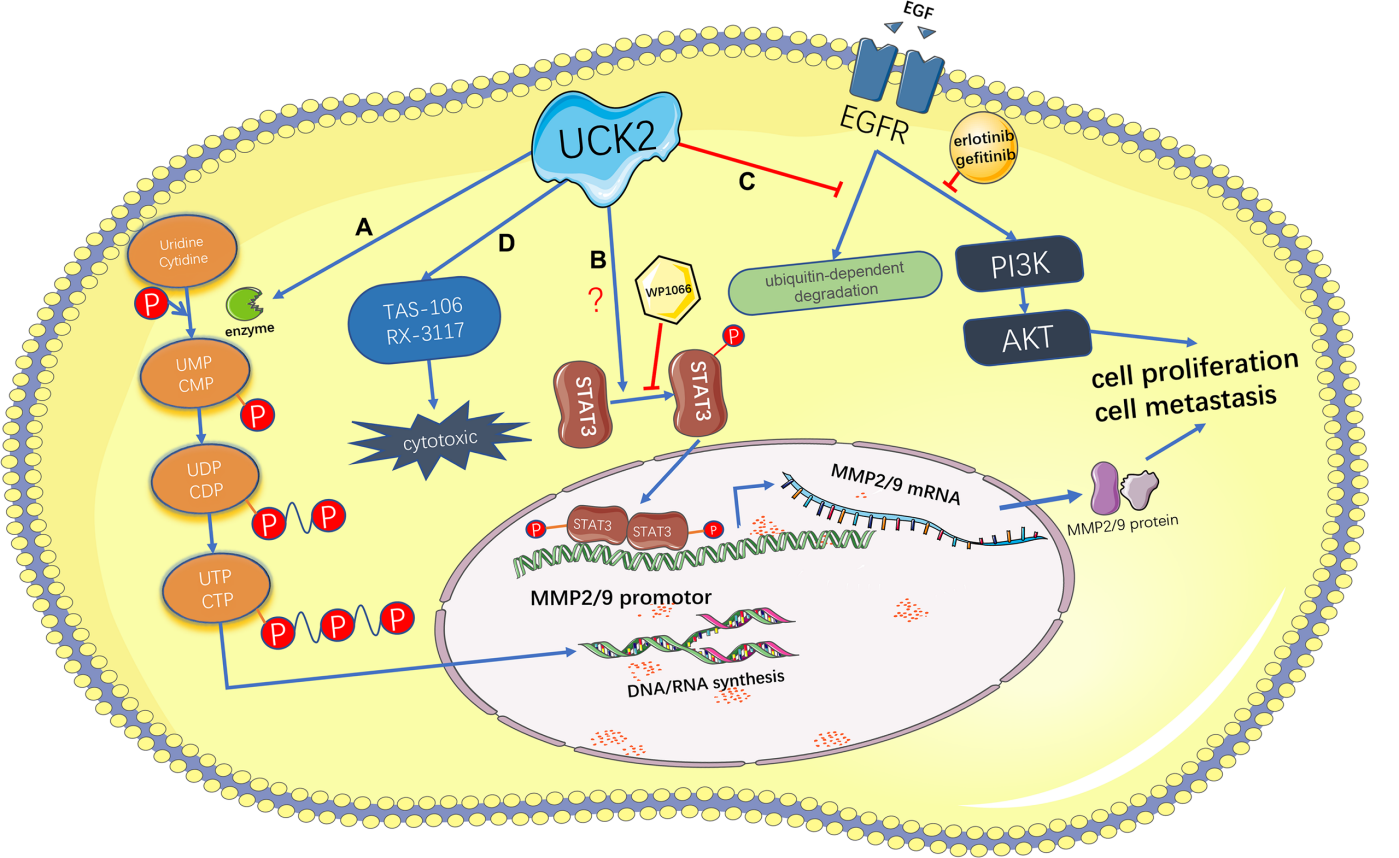
Schematic representation of the catalytic-dependent and -independent features of UCK2 and the therapeutic potential of UCK2 in cancer treatment. **(A)** As a rate-limiting enzyme of the pyrimidine salvage synthesis pathway, UCK2 phosphorylates uridine and cytidine to uridine monophosphate (UMP) and cytidine monophosphate (CMP), respectively. **(B)** UCK2 can activate the STAT3-MMP2/9 axis to promote tumor cell proliferation and metastasis, but the underlying mechanism remains elusive. This non-catalytic function of UCK2 can be inhibited by a STAT3 inhibitor, WP1066. **(C)** By inhibiting EGF-induced EGFR ubiquitination and degradation, UCK2 can activate EGFR-AKT pathway to promote tumor cell proliferation and metastasis. This non-catalytic function of UCK2 can be inhibited by EGFR inhibitors: erlotinib and gefitinib. **(D)** UCK2 can be utilized to catalyze cytotoxic ribonucleoside analogs, such as TAS-106 and RX-3117, for cancer chemotherapy.

The mechanisms by which UCK2 promotes tumor development may be mainly attributed to its metabolic function by providing sufficient nucleotides to support enhanced DNA and RNA synthesis in tumor cells (**Figure 3**). For example, direct UCK2 inhibition induced cell death in colorectal cancer cells by reducing 18S RNA expression and led to subsequent cell cycle arrest (24), suggesting that UCK2 inhibition could impair RNA biosynthesis. Meanwhile, UCK2 inhibition will lead to defects of ribosomal biogenesis and nucleolar stress. In response to nucleolar stress, the ribosomal proteins will release from nucleolus and bind with MDM2. This blocks MDM2-mediated p53 ubiquitination and results in stabilization and activation of p53 and p53-mediated apoptosis (24). In addition to the metabolic role, our and other groups have identified the non-metabolic roles of UCK2 in tumor progression (**Figure 3**), especially in HCCs (26, 27). For example, UCK2 has been shown to promote the *in vitro* migration and invasion and the *in vivo* metastasis by activating the STAT3-MMP2/9 signaling axis in HCCs (26). We demonstrated that unlike UCK2 wild type, overexpression of catalytic dead UCK2 mutant (UCK2^D62A^) failed to promote HCC cell proliferation but still enhanced HCC metastasis, suggesting that UCK2 not only could promote HCC cell proliferation in a catalytic dependent manner but also could facilitate HCC metastasis in a catalytic independent manner (27). We further demonstrated that UCK2 could interact with EGFR to block EGF-induced EGFR ubiquitination and degradation and, in turn, activate the EGFR-AKT signaling pathway (27) (**Figure 3**). In contrast to UCK2 protein that plays an oncogenic role in tumor progression, a recent study suggested that UCK2 could generate circular RNAs, which may play a tumor suppressive role. CircUCK2 (hsa_circ-001128 and hsa_circ_001357) could serve as a sponge of miR-767-5p to relief its inhibition on TET1, resulting in reduced cell proliferation and invasion in enzalutamide-resistant prostate cancer cells (47).

## Targeting UCK2 Activity in Anti-Tumor Therapy

In clinical oncology, the capacity of UCKs in phosphorylating cytidine and uridine has been employed to activate several cytotoxic ribonucleoside analogs, such as TAS-106 (1-[3-C-ethyl-β-ribo-pentofuranosyl/cytosine, ECyd]) (25, 41, 42), 1-(3-C-ethynyl-beta-D-ribopentofuranosyl)uridine (EUrd) (42), 5-fluorouracil (5-FU) (43), and RX-3117 (fluorocyclopentenylcytosine) (44, 45), for cancer chemotherapy. Compared to UCK1 that has lower catalytic activity and universal expression pattern in normal tissues, UCK2 is the ideal therapeutic target due to its greater catalytic activity and exclusive and abundant expression in tumors (48). In fact, some previous studies have demonstrated close association between tumor UCK2 levels and objective response to those cytotoxic ribonucleoside analogs. For example, the protein and mRNA levels of UCK2 but not UCK1 were closely associated with TAS-106 phosphorylation and cellular sensitivity to TAS-106 (25); loss-of-function mutations of *UCK2* but not *UCK1* were observed in cancer cells resistant to either ECyd or EUrd (42, 49); knockdown and upregulation of *UCK2* were related to 5-FU resistance and sensitization respectively in colorectal cancer cells (43, 50); UCK2 but not UCK1 was responsible for activation of RX-3117 (48); UCK2 could serve as a prospective biomarker of potential response to RX-3117 treatment in pancreatic cancer patients (34). In addition to the metabolic feature of UCK2 which has been employed for cancer treatment, our recent study has demonstrated that UCK2 can non-metabolically activating EGFR-AKT signaling to promote HCC progression, which may provide novel UCK2-based therapeutic strategies for cancer treatment. In fact, we have demonstrated that concurrent pharmacologic targeting the metabolic and non-metabolic features of UCK2 by ECyd and Erlotinib, respectively, could synergistically inhibit HCC growth and metastasis (27).

## Summary and Discussion

The salvage nucleotide synthesis plays an important role in tumor development by providing sufficient nucleotides for rapid tumor cell proliferation. As a rate-limiting enzyme of the pyrimidine salvage synthesis pathway, *UCK2* is exclusively and abundantly expressed in human placenta and many types of solid and hematopoietic cancers and closely associated with poor prognosis in many malignancies including HCCs, pancreatic cancer, and lung cancer. According to the recent studies of our and other groups in HCCs, we believe that UCK2 may promote tumor progression in both catalytic dependent and independent manners. On one hand, UCK2 catalyzes sufficient nucleotides to support tumor proliferation; on the other hand, UCK2 activates several oncogenic pathways, such as STAT3-MMP2/9 and EGFR-AKT, to promote tumor progression in a catalytic independent manner. By employing the catalytic feature of UCK2, several cytotoxic nucleoside analogs have been developed for cancer chemotherapy. Meanwhile, more attention needs to be paid to the catalytic independent feature of UCK2 in promoting tumor progression, because our recent study has demonstrated that concurrent pharmacologic targeting the catalytic and non-catalytic features of UCK2 could synergistically inhibit HCC growth and metastasis (**Figure 3**).

## Author Contributions

YF and XW drafted this manuscript. YF, XW, LG, KW, JL, YM, and XK performed extensive literature search and discussion. YT and HW conceived the idea and edited the manuscript. All authors contributed to the article and approved the submitted version.

## Conflict of Interest

The authors declare that the research was conducted in the absence of any commercial or financial relationships that could be construed as a potential conflict of interest.

## Publisher’s Note

All claims expressed in this article are solely those of the authors and do not necessarily represent those of their affiliated organizations, or those of the publisher, the editors and the reviewers. Any product that may be evaluated in this article, or claim that may be made by its manufacturer, is not guaranteed or endorsed by the publisher.

## Abbreviations

AKT, serine/threonine kinase; CMP, cytidine monophosphate; CRC, colorectal carcinoma; CRPC, castration-resistant prostate cancer; CTP, cytidine triphosphate; EC, endometrial cancer; EGFR, epidermal growth factor receptor; GWASs, genome-wide association studies; HCC, hepatocellular carcinoma; LncRNA, long non-coding RNA; MDM2, MDM2 proto-oncogene; MMP2, matrix metallopeptidase 2; MMP9, matrix metallopeptidase 9; NMP, nucleoside monophosphate; OS, overall survival; p53, tumor protein p53; STAT3, signal transducer and activator of transcription 3; TET1, tet methylcytosine dioxygenase 1; TNM, tumor, node and metastasis; UCK1, uridine-cytidine kinase 1; UCK2, uridine-cytidine kinase 2; UMP, uridine monophosphate; UTP, uridine triphosphate.
